# Quality of Life among Iranian Postmenopausal Women: A Systematic Review and Meta-Analysis

**DOI:** 10.31661/gmj.v9i0.1649

**Published:** 2020-03-04

**Authors:** Khadijeh Sharifi, Zahra Tagharrobi, Zahra Sooki

**Affiliations:** ^1^Trauma Nursing Research Centre, Faculty of Nursing and Midwifery, Kashan University of Medical Sciences, Kashan, Iran

**Keywords:** Menopause, Quality of Life, Systematic Review, Meta-Analysis

## Abstract

Menopause can cause mental, physical, vasomotor, and sexual symptoms and problems, which negatively affect the quality of life (QOL). The aim of this study was to systematically evaluate QOL among Iranian postmenopausal women. This systematic review was conducted on cross-sectional studies that were published between 2000 and 2018. An online search to find studies published in English or Persian was conducted in the databases of Web of Science, PubMed, ScienceDirect, Scopus, Google Scholar, Scientific Information Database, Magiran, and IranMedex. Search key terms were "quality of life", "menopause", and "Iran". Fourteen studies were eligible for this study. The Strengthening the Reporting of Observational Studies in Epidemiology (STROBE) checklist was used for quality appraisal. The mean and standard deviation of QOL and its domains were extracted from the selected studies. Study data were analyzed using the Review Manager (v. 5.0) and the STATA (v. 12.0) software. The mean of total QOL among 3413 postmenopausal women was 57.89±12.8 (in the possible range of 0–174). The means of its vasomotor, psychosocial, physical, and sexual domains were 7.86±2.14, 19.43±2.05, 40.58±3.33, and 6.71±1.77, respectively. The QOL among Iranian postmenopausal women is nearly higher than the moderate level. The lowest and the highest levels of QOL are related to the physical and sexual domains, respectively. Health authorities need to develop educational interventions to promote postmenopausal women’s QOL, particularly in the physical domain.

## Introduction

As a physiological phenomenon, menopause is the termination of menstruation and reproduction. It is among the most important phases of women’s life [1]. Life expectancy improvement in recent years has significantly increased the number of postmenopausal women [2]. Currently, women spend around one third of their lives after menopause and hence, postmenopausal period has received special attention during recent years. Menopause is associated with different physiological changes and problems such as hot flashes, nighttime sweating, anxiety, headache, fatigue, irritability, and sleep disorders [3,4]. Thereby, it can significantly affect quality of life (QOL)[1,4,5]. According to the World Health Organization, QOL is “an individual’s perception of their position in life in the context of the culture and value systems in which they live and in relation to their goals, expectations, standards, and concerns” [6,7]. QOL includes different vasomotor, sexual, physical, and mental aspects and its improvement is among the goals of the health for all programs[8]. Different studies reported the negative effects of menopause on QOL [5,9-13]. For instance, a study reported significant correlations between menopausal symptoms and different aspects of QOL and concluded that the climacteric period can have negative effects on the physical and mental aspects of QOL [10]. Another study also found that postmenopausal women had poor QOL [14]. Progression of menopause was also reported to reduce QOL, so that older postmenopausal women had lower QOL [15]. Contrarily, some studies reported the non-significant effects of menopause on QOL [16,17]and the non-significant correlations of menopausal symptoms with QOL [17-20]. A review study also reported that there is no clear pattern for the relationship between menopause and the mental aspect of QOL [20]. QOL measurement could provide valuable data about patients’ feelings, problems, and needs and also about the effects of preventive and health-promoting measures and programs[13].However, previous studies reported varying levels of QOL among menopausal women [4,5,9-13]and hence, there is no conclusive evidence about their QOL status. To fill this gap, this study was conducted. The aim of the study was to systematically evaluate QOL among Iranian postmenopausal women.

## Search Strategy

 As a systematic review and meta-analysis, this study was conducted in 2018 on studies into QOL among Iranian postmenopausal women.

 An online search was performed in national and international scientific databases such as Web of Science, PubMed, ScienceDirect, Scopus, Google Scholar, Scientific Information Database, Magiran, and Iranmedex. Search key terms were “quality of life”, “life quality”, “health-related quality of life”, “HRQOL”, “menopause”, and “Iran”as well as their equivalents in the Medical Subject Headings. Boolean operators “AND” and “OR” were also used to combine search results. Since some Iranian databases showed no sensitivity to search operators (AND, OR), the search in the Iranian database was done by main, sensitive, and public key­words such as “menopause” and “quality of life” to provide a high sensitivity.

###  Study Selection

**Figure 1:**
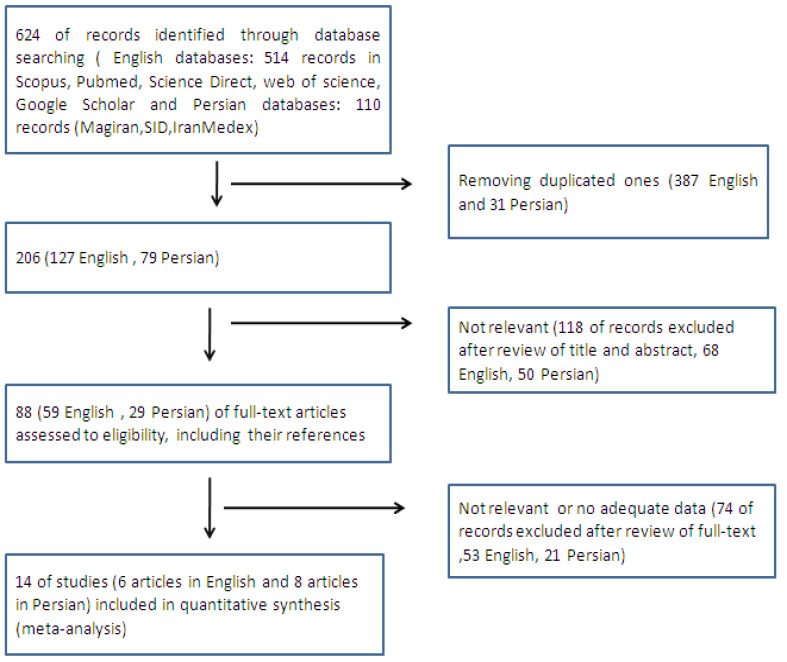
The flow diagram of the study

Study selection was independently done by two of the authors(Z.S. and Kh.Sh.) and their disagreements were resolved by a third author (Z.T.). In the first step of theonline search, 624 records were retrieved. Similar records were excluded and 206 records were kept. In the second step, irrelevant studies were excluded and 88 studies which were in some ways relevant to menopausal women’s QOL were extracted. In the third step, all these 88 studies and their references were assessed for eligibilityand finally, 14 eligible studies were selected for meta-analysis (Figure-1). Eligibility criteria were publication in Persian or English between 2000 to July 2018, cross-sectional descriptive design, and QOL assessment among postmenopausal women using the Menopause-specific Quality of Life (MenQOL) questionnaire. Any ambiguities in the selected studies were clarified throughcontacting their authors viaemail or telephone. The EndNote software (v. X7, Thomson Reuters EndNote) was used to save and manage the studies. The 29-item MenQOLassesses postmenopausal women’s QOL in four main domains, namely vasomotor (3 items), psychosocial (7 items), physical (16 items), and sexual (3 items). Its items are scored from 0 (“Lowest severity”) to 6 (“Highest severity”). Therefore, the total score of the questionnaire and its vasomotor, psychosocial, physical, and sexual domains can range from 0 to respectively 174, 18, 42, 96, and 18. Higher scores show lower QOL and vice versa. The scoring of MenQOL items in the selected studies had been performed on either a 0–5 or 0–6 scale[21]. In the present study, the results reported in the retrieved studies were changed into a 0–6 scale.

###  Quality Appraisal

**Figure 2:**
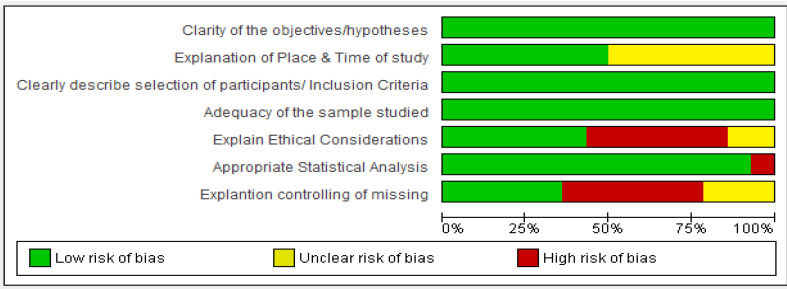
The total risk of publication bias

 Quality appraisal was performed using the Strengthening The Reporting of Observational Studies in Epidemiology (STROBE) checklist. The seven main criteria assessed using this checklist were;detailed explanation of the purpose, accurate description of the place and the time of the study, inclusion criteria and selection procedures, adequacy of sample size, ethical considerations, statistical analysis, and control of lost samples. Each of the criteria has scored; high 0, unclear 1 and low risk of bias 2.The more score, the lower risk (Table-1 and Figure-2).

###  Data Analysis

**Figure 3:**
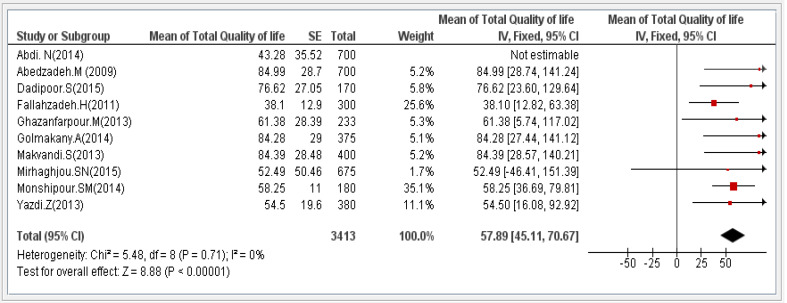
The mean score of total QOL in each study based on the results of the fixed-effects model

**Figure 4:**
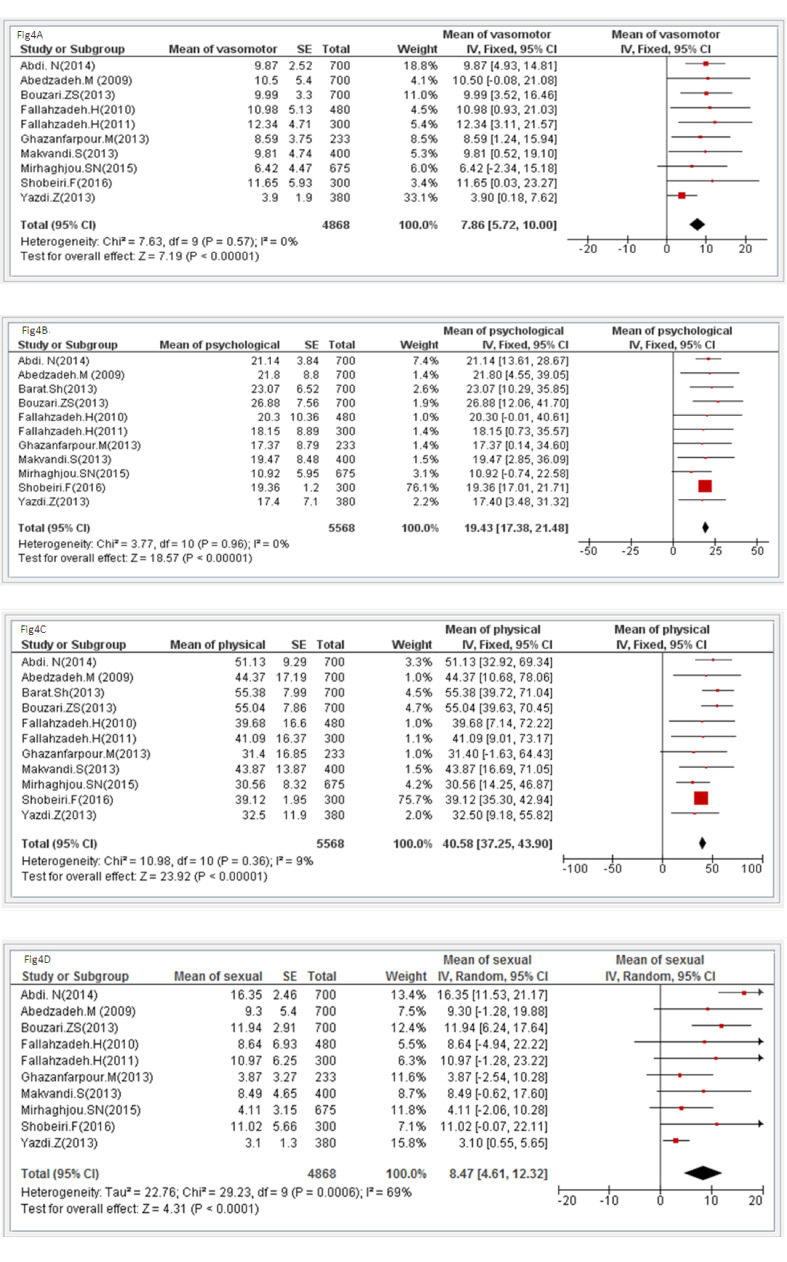
The mean scores of QOL domains in each study based on the results of the fixed-effects model

Study data were analyzed at a significance level of less than 0.05 using the Review Manager (v. 5.0, Cochrane community, London, UK) and the STATA (v. 12.0, Stata Corp, College Station, TX, USA) software. Heterogeneity was tested through the Q test and the I2statistic [22]. Meta-analysis was performed through the randomeffects model for sexual domain (because of high heterogeneity) and for other variables the fixed effects model (because of nothing heterogeneity, Figures-3 and 4). The Tau squared value was also used to assess the variance among the studies[23] and publication bias was assessed through Egger’s test. The means and standard deviations of QOL and its four domains were extracted from each study and then, were combined proportional to the sample size of each study using the fixed effects model.

## Results

**Figure 5:**
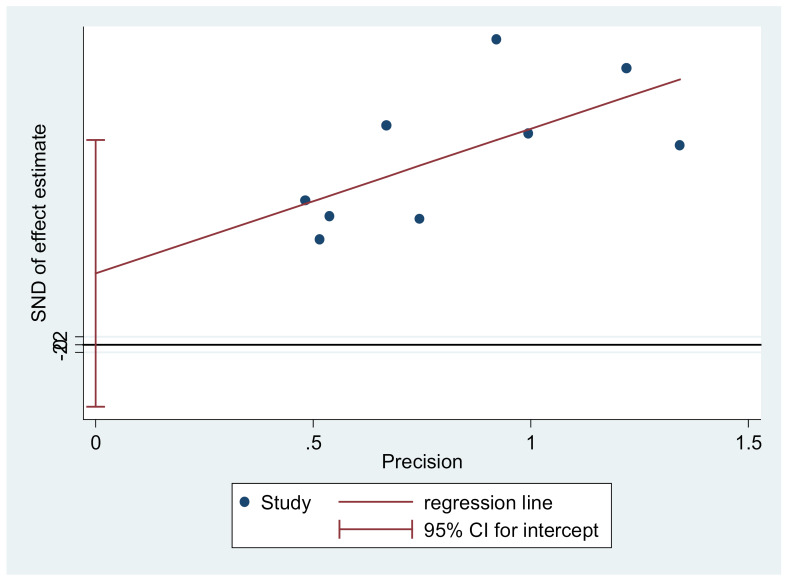
The Egger test for publication bias (total quality of life),P=0.247

**Figure 6:**
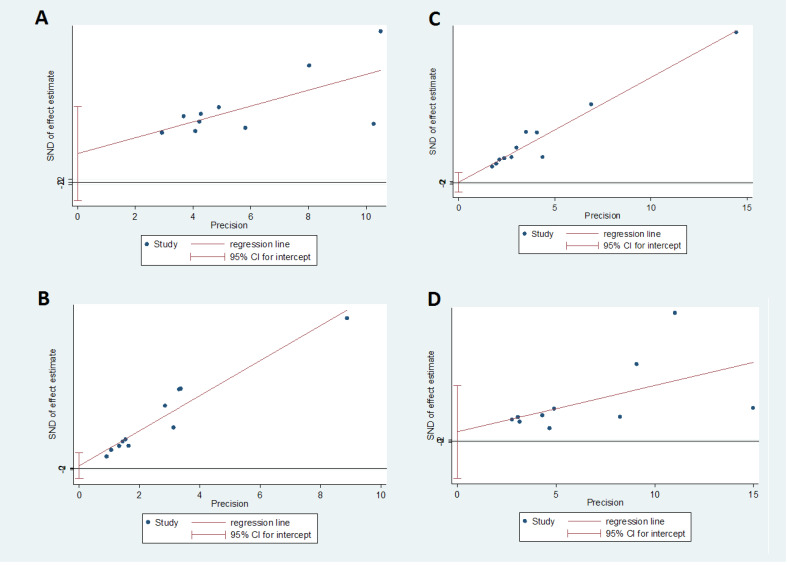
The Egger test for publication bias **A**: Vasomotor domain (P=0.198); **B**: Psychological domain (P=0.907); **C**: Physical domain (P=0.608); **D**: Sexual domain (P=0.659)

The total number of postmenopausal women in the fourteen reviewed studies was 6293. The mean of their age was 54.33±2.58. Table-2 shows the characteristics of the reviewed studies. The Egger’s test showed that there was no publication bias (Figures-5 and 6). The mean of total QOL had been reported only in ten studies with a total sample size of 3413. The mean of total QOL in these ten studies was calculated to be 57.89 (95% confidence interval: 45.11–70.67). Figures-3 and 4 show the total mean scores of QOL and its different domains, respectively. The lowest total mean score of QOL among the reviewed studies was 38.1 (95% confidence interval [95%CI] 12.82–63.38) [21], while the highest total mean score of QOL was 84.99 (95% CI: 28.74–141.24)[14].

## Discussion

This study aimed to estimate QOL among Iranian postmenopausal women. In total, fourteen studies were reviewed. However, the total number of studies analyzed in meta-analysis varied for each of MenQOLdomains. Accordingly, the number of the analyzed studies was ten for the vasomotor and the sexual domains, eleven for the psychological and the physical domains, and ten for total QOL. The mean score of the vasomotor domain of QOL was 7.86±2.14 (in the possible range of 0–18), which is almost at moderate level. This value was 9.6±6.6 in a cross-sectional study on 2703 American postmenopausal women [13], 5.49±4.74 in a study on 770 Iranian premenopausal women aged 45–60 [24], and 8.88±8.82 in a cross-sectional study on 100 Indian postmenopausal women [25]. The difference among these studies respecting the mean score of the vasomotor domain of QOL can be attributed to the fact that QOL is affected by factors such as age [10,26-28], ethnicity, sociocultural environment, lifestyle [13,15], educational level, employment, income, physical activity, and access to reliable information services for receiving counseling and information about coping with menopause [29,30]. The mean score of QOL in the psychosocial domain was 19.43±2.05 (in the possible range of 0–42).This score shows moderate QOL in the psychosocial domain. This value in two previous studies was 23.1±12.6 [13] and 26.64±17.88 [25]. Irrespective of menopause, low QOL may be associated with mood changes and the feeling of tension [31]. In a review study by Vesco *et al*. (2008), nine reviewed studies had reported that menopause had no significant correlations with depression, negative mood, major depressive disorder, general mental health, and other mental symptoms, while three studies had found higher prevalence of depression among postmenopausal women and one study had reported higher well-being among postmenopausal women. That study also reported that socioeconomic status and educational level could affect the severity of menopausal symptoms and QOL [20]. Lifestyle factors such as diet, physical exercise, and social activities can affect psychosocial status and QOL [32]. Some studies on postmenopausal women in Iran also showed that women with membership and participation in social association had better QOL and positive emotionsprobably due to governmental support for these associations [33,34]. Also, our findings revealed that the mean score of QOL in the physical domain was 40.58±3.33 (in the possible range of 0–96). This score denotes that Iranian postmenopausal women’s QOL in the physical domain was better than the moderate level. This value in two earlier studies was 56±24 [13]and 63.54±35.34 [25]. A review study showed that premenopausal period is associated with high levels of physical symptoms [35]. A cross-sectional study on 410 postmenopausal women in Turkey also indicated that they had experienced physical symptoms more frequently than the symptoms in other domains of QOL [36], while a study reported lower physical QOL at the beginning of menopause[15]. Another cross-sectional study in Malaysia on 258 Malay, Indian, and Chinese postmenopausal women showed that Asian women had mostly experiencedmenopause-related musculoskeletal symptoms, while western women had mostly experienced vasomotor symptoms. These findings imply the effects of ethnicity on menopausal symptoms. Of course, that study highlighted that the effects of ethnicity on menopausal symptoms are still questionable [37]. We also found that the mean score of QOL in the sexual domain was 6.71±1.77 (in the possible range of 0–18), denoting high QOL. Two previous studies reported that the mean score of sexual QOL among postmenopausal women was 8.7±6.3 [13] and 6.9±8.58 [25]. Another study reported that the mean score of sexual QOL was 9.3±6.6 in early menopause and 10.77±7.5 in late menopause, denoting QOL reduction with age[15]. These contradictory results among the studies are due to differences in their samples, designs, and sociocultural contexts [38].Menopause-related hormonal changesprofoundly affect renal function and cause symptoms, which considerably affect QOL and sexuality. Therefore, sexual dysfunction is among the most common problems during menopausal transition and postmenopausal period. The most common sexual complaints among postmenopausal women are vaginal dryness, dyspareunia, and decreased libido. Therefore, regular sexual assessment should be performed for middle-aged and older women and they should be provided with the opportunity to verbalize their sexual problems. Such assessments can facilitate the early diagnosis and management of sexual dysfunction[39]. Study findings also showed that the total mean score of QOL among 3413 postmenopausal women was 57.89±12.8 (in the possible range of 0–174), indicating good QOL. Several earlier studies in different countries also reported good QOL among postmenopausal women [40-42]. However, some studies reported low QOL among menopausal women[14,43,44]. Menopause, aging, social deprivation, and affliction by chronic illnesses can negatively affect QOL amongmiddle-aged and older women. Some other studies found no significant relationship between menopause and QOL. For instance, a study with an eight-year follow-up period on 1165 Finnish women aged 45–64 found that menopause had no significant relationship with QOL [45]. A cross-sectional study in Spain and Majorca on 378 postmenopausal women and fifty pre-menopausal women also found a weak relationship between menopause and QOL [46]. Another study in Hong Kongon women aged 40-59 years also showed no significant difference between premenopausal and postmenopausal QOL [19]. These contradictory results may be due to the fact that although menopausal symptoms may affect QOL of most women[36,37], only a small number of them experiencethe sever effects of menopause [37]. Another justification for these contradictory results is the fact that women gradually learn how to manage and cope with menopausal symptoms. In other words, QOL in menopausal and postmenopausal periods is affected by women’s ability to cope with menopausal symptoms and menopause-related changes in the body as well as their feelings of satisfaction and happiness in these periods. Studies have shown the positive effects of education about positive thinking, physical activity, and lifestyle modification on menopausal symptoms and QOL [6,19]. Therefore, educational interventions are recommended for QOL improvement among postmenopausal women.

###  Study Limitations

 This study reviewed only cross-sectional studies and hence, provides no information about the effects of the normal process of aging on postmenopausal women’ QOL. Moreover, at the time of the study, online scientific databases in Iran provided no option for combining search results using Boolean operators “AND” and “OR”.

###  Strengths

 This study reviewed studies that had used a valid and reliable MenQOL questionnaire.

## Conclusion

 This systematic review and meta-analysis show that Iranian postmenopausal women’s QOL is higher than moderate. The lowest and the highest quality of life are related to the physical and sexual domains, respectively. Policymaking for public education, patient education, and healthcare staff education are recommended to facilitate QOL improvement among postmenopausal women.

## Conflict of Interest

 The authors declare that they have no conflict of interest.

**Table 1:** TheRisk of Publication Bias in Each Study

**Author**	**Clarity of the objectives/ hypotheses**	**Explain place and time of the study**	**Clarity of inclusion criteria and selection procedures**	**Adequacy of sample size**	**Explain ethical considerations**	**Appropriate statistical analysis**	**Explain on controlling of missing**	**The risk of publication bias in each study**
** Abdiet al. [47] **	2	2	2	2	0	2	0	10
**Abedzadehet al[ 14]**	2	1	2	2	2	0	0	9
** Baratet al. [26] **	2	1	2	2	0	2	2	11
** Bouzariet al. [9] **	2	1	2	2	2	2	0	11
** Dadipooret al. { Daipoor, 2015 #146}[48] **	2	1	2	2	1	2	1	11
** Fallahzadehet al. [11] **	2	2	2	2	0	2	2	12
** Fallahzadehet al. [21] **	2	1	2	2	0	2	0	9
** Ghazanfarpouret al. [49] **	2	1	2	2	2	2	2	13
** Golmakanyet al. [27] **	2	2	2	2	0	2	0	10
** MaKvandiet al. [50] **	2	2	2	2	0	2	2	12
** Mirhaghjouet al. [51] **	2	2	2	2	2	2	2	14
** Monshipooret al. [28] **	2	2	2	2	2	2	0	12
** Shobeiri et al. [29] **	2	2	2	2	2	2	1	13
** Yazdiet al. [52] **	2	1	2	2	1	2	1	11

**Table 2:** The Characteristics ofthe Reviewed Studies

**Author**	**Publication Year**	**Sample Size**	**City**	**Age** **( Mean±SD )**	**Quality of life and its domains (adjusted findings)**				
					**Vasomotor** **( Mean±SD )**	**Psychological** **( Mean±SD )**	**Physical** **( Mean±SD )**	**Sexual** **( Mean±SD )**	**Total Quality of Life** **( Mean±SD )**
** Abdiet al. [47] **	2014	700	Tehran	59.64 **±**2.32	9.87±2.52	21.14±3.84	51.13±9.29	16.35±2.4	43.28±35.52
** Abedzadehet al. [14] **	2009	700	Kashan	53.8 **±**4.25	10.5±5.4	21.8±8.8	44.37±17.19	9.3±5.4	84.99±28.7
** Baratet al. [26] **	2013	700	Babol	48.57 **±**1.55	-	23.07±6.52	55.38±7.99	-	-
** Bouzariet al. [9] **	2013	700	Babol	52.92 **±**2.5	9.99±3.3	26.88±7.56	55.04±7.86	11.94±2.91	-
** Dadipooret al. { Daipoor, 2015 #146}[48] **	2015	170	Bandar Abbas	57 **±**8.15	-	-	-	-	76.62±27.05
** Fallahzadehet al. [11] **	2010	480	Yazd	55.68 **±**6.22	10.98±5.13	20.3±10.36	39.68±16.6	8.64±6.93	-
** Fallahzadehet al. [21] **	2011	300	Yazd	53.9 **±**4.9	12.34±4.71	18.15±8.89	41.09±16.37	10.97±6.25	38.1±12.9
** Ghazanfarpouret al. [49] **	2013	233	Shiraz	54 **±**5.1	8.59±3.75	17.37±8.79	31.40±16.85	3.87±3.27	61.38±28.39
** Golmakanyet al. [27] **	2014	375	Neyshabur	55.4 **±**5	-	-	-	-	84.28±29
** MaKvandiet al. [50] **	2013	400	Ahvaz	54.81 **±**5.66	9.81±4.74	19.47±8.48	43.87±13.87	8.49±4.65	84.39±28.48
** Mirhaghjouet al. [51] **	2015	675	Rasht	55.24 **±**4.51	6.42±4.47	10.92±5.95	30.56±8.32	4.11±3.15	52.49±50.46
** Monshipooret al. [28] **	2014	180	Rasht	60.57 **±**7.5	-	-	-	-	58.25±11
** Shobeiriet al. [29] **	2016	300	Hamadan	55.46 **±**5.49	11.65±5.93	19.36±1.20	39.12±1.95	11.02±5.66	-
** Yazdiet al. [52] **	2013	380	Qazvin	57.6 **±**6.02	3.9±1.9	17.4±7.1	32.5±11.9	3.1±1.3	54.5±19.6
